# MicroRNA-18 facilitates the stemness of gastric cancer by downregulating HMGB3 though targeting Meis2

**DOI:** 10.1080/21655979.2022.2062529

**Published:** 2022-04-13

**Authors:** Yingjun Zhang, Weijian Lin, Weiping Jiang, Zhenfa Wang

**Affiliations:** a Oncology Department of Radiotherapy, Zhongshan Hospital of Xiamen University, Xiamen, Fujian China; b Department of Gastrointestinal Surgery, Zhongshan Hospital of Xiamen University, Xiamen, Fujian, China

**Keywords:** MicroRNA-18, meis2, HMGB3, gastric cancer cell stemness

## Abstract

The recurrence and metastasis of gastric cancer are related to the stemness of gastric cancer cells. Researches have shown that miR-18 level is negatively correlated to the occurrence and development of certain cancer types. However, the effects of miR-18 on the stemness of gastric cancer remain uncertain. In this research, gastric cancer cell lines with stable overexpression of miR-18 were constructed through lentivirus infection. CCK-8 assay, RT-qPCR, Western blot, flow cytometry, and *in vivo* tumorigenesis assays were performed to evaluate the effects of miR-18 on the stemness of gastric cancer cells. Moreover, luciferase reporter assays found that Meis2 was the target of miR-18. Furthermore, we also found that the low-expressed oncogene HMGB3 is involved in this miR-18/Meis2 axis to further promote the stemness of gastric cancer cells. These findings suggest that the miR-18/Meis2/HMGB3 axis may be potential prognostic indicators for patients with gastric cancer.

## Graphical Abstract

**Figure:**
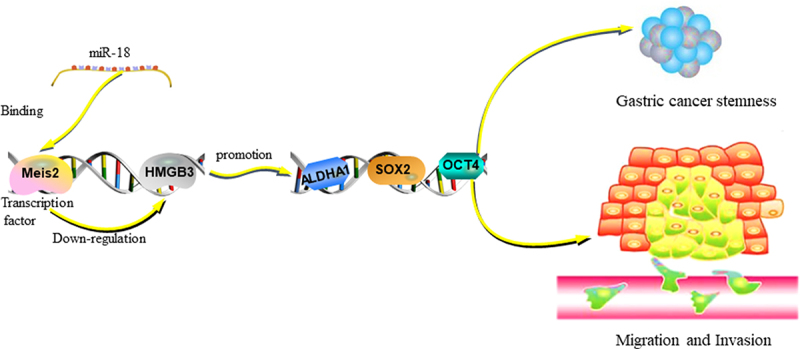

## 1. Introduction

With the development of medical technology for decades, the incidence and mortality of gastric cancer are yearly declined [1]. However, gastric cancer (GC) is still the third leading cause of cancer death in the world. GC belongs to a disease of the digestive system, and its developing mechanisms are diverse and complex [2]. Therefore, there is an urgent need to explore the pathogenesis of GC and find novel targets to improve the diagnosis and treatment options.

Cancer stem cells (CSCs) are proposed to be a small number of tumor cells with characteristics of stem cells and are responsible for tumor progression and initiation [3]. CSCs have complex heterogeneity and unlimited proliferation ability, which leads to tumor proliferation, metastasis, recurrence and drug resistance [4]. Therefore, it will help us to find new targets for effective treatment by revealing the molecular mechanism of CSCs formation and the relationship between CSCs and drug resistance.

In the past few decades, microRNAs (miRNAs), approximately 19–22 nucleotides in length, are endogenous non-coding small RNAs that are evolutionarily conserved, and participate in multiple cellular biological processes including proliferation, differentiation, and cell apoptosis [5–7]. MiRNAs are ubiquitous in eukaryotic cells, bind to the 3’-untranslated (3’UTR) of the target mRNAs, and thus regulate gene expression at the post-transcriptional level [8]. The abnormal expression of miRNAs is closely related to the occurrence and development of tumors [9]. For instance, miR-375 induces docetaxel resistance in prostate cancer by targeting SEC23A and YAP1 [10]. In breast cancer, miR-375 not only inhibits the stemness of breast cancer cells, but also reduces the adriamycin resistance of adriamycin resistant MCF-7^Adr^ cells by impeding the JAK2/STAT3 signaling pathway [11]. MiR-148a suppresses the stemness of colorectal cancer cells by regulating the WNT and β-catenin signaling pathways, increasing chemotherapy sensitivity, cell invasion, and migration [12]. MiR-616-3p promotes GC angiogenesis through the PTEN/AKT pathway [13]. MiR-18 acts as an oncogene which belongs to the miR-17-92 cluster and its carcinogenic effect has been fully documented [14]. Overexpression of this cluster is involved to accelerated tumor growth and cell proliferation [15]. MiR-18 has been reported to be engaged in the progression of various solid tumors, including nasopharyngeal carcinoma [16], GC [17], ovarian cancer [18], colorectal cancer, and pancreatic cancer [19,20]. Previous studies have found that miR-18 was one of the most up-regulated miRNAs in GC through gene microarray profiling the expression of 847 miRNAs in GC from Chinese patients. A high level of miR-18a can be detected in GC patients compared to normal individuals; Upon removal of the tumor, miR-18 level was reduced significantly [21]. Notably, Chen’s et al. study found that miR-18 regulates P53 expression by directly targeting IRF2, which has a high predictive value for the prognosis of GC patients [17]. However, the mechanism of miR-18 regulating GC stemness is still unclear.

In the current study, bioinformatics analysis predicted that Meis Homeobox 2 (Meis2) is the target gene of miR-18. In addition, TCGA database analysis found that Meis2 was downregulated in GC. Meis homeodomain transcription factors appertain the TALE, and are composed of three human homologues Meis1, Meis2, and Meis3, which can participate in the transformation of human embryonic stem cells from endothelial cells to hematopoietic cells [22]. Recently, it has been reported that Meis2 can regulate the chemotherapy resistance of CRC stem cells by regulating the level of promoter methylation [23,24]. Furthermore, studies have found that Meis2 holds carcinogenic effects in the development of neuroblastoma, leukemia, bladder cancer, prostate cancer, and ovarian cancer [25]. Moreover, Sadegh Saghafinia et al. have demonstrated that miR-181 induced the IT to MLP transition by targeting Meis2, leading to the up-regulation of the developmental transcription factor HMGB3 in pancreatic neuroendocrine tumors [26]. HMGB3, as a gene promoter with a significantly high score, belongs to the x-linker member of the HMG family which is closely related to the poor prognosis of gastric cancer patients [27]. Guo et al. have proved that HMGB3 is involved in gene transcription, DNA repair, and genome stability, and its expression is correlated with the invasion, metastasis, and poor prognosis in multiple cancers [28].

In this study, we aimed to explore the mechanism of miR-18 stemness and its biological function in gastric cancer cells. miR-18 was found to promote the stemness of GC cells through *in vivo* and *in vitro* experiments. Integrate TCGA database with experimental verification, we identified Meis2 as a direct target of miR-18. We further demonstrated that miR-18 promoted the stemness of GC by targeting Mesi2 to up-regulate HMGB3.

## 2 Materials & Methods

### Mammalian cell culture and chemical reagent

Three GC cell lines (AGS, MKN45 and MGC803) were obtained from ATCC. Normal gastric epithelial cell line GES-1 was preserved in our laboratory. GC cell lines were cultured in 1640 medium (11,879,020, Thermo, USA), supplemented with 10% fetal bovine serum (10101145C, Thermo, Australian, USA), 100 units/mL penicillin and 0.08 mg/mL streptomycin. All cells were incubated in a 5% CO_2_ atmosphere at 37°C.

### Plasmid transfection, miR-18 mimics, inhibitor

Cells were planted on microplates to achieve a growth density of 40–50%, then cells were transfected using Transfect-Mate transfection reagent (GenePharma, Shanghai, China) following the Transfect-Mate transfection reagent instruction. miR-18 mimics, inhibitor, and miR-18^NC^ were synthesized by GenePharma Biotechnology (Shanghai, China) [29].

### CCK8 assays

CCK-8 was used to examine cell viability and chemotherapy sensitivity. GC cell lines were seeded into 96-well plates at a density of 3000–5000 cells/well, and the drug-loaded medium was replaced 24 h later and incubated at 37°C, 5% CO_2_ for 1 days, 2 days, 3 days, respectively. CCK8 was added into the 96 – well plates. Finally, multifunctional microplate reader (Bio-Rad, Hercules, CA, USA) was used to measure the absorbance at 450 nm [11].

### Construction and screening of stable overexpression miR-18 cell lines

Stable overexpression AGS^NC-^^GFP^, AGS^18-^^GFP^, MKN-45^NC-^^GFP^, MKN-45^18-^^GFP^ cell lines were acquired by lentivirus infection. And then the positive cell lines were screened with puromycin according to manufacturer’s protocols (HANBIO, Shanghai, China). Cells were performed with fluorescence microscope to observe the changes in fluorescence intensity after infection 3 days, observing the cell status and then subjected to puromycin (Sigma, 2 μg/ml) screening at 2 weeks. Lentivirus infection efficiency was detected by QRT-PCR [30].

### Reporter vector construction and luciferase reporter assay

The detailed experimental protocol was performed as we referred before [31]. Luc-Meis2 ^3’ UTR-^^WT^ (including binding site of miR-18) and Luc-Meis2 ^3’ UTR-^^MUT^ (including mutated binding sites of miR-18) were constructed employing the Fast Mutagenesis Kit V2 (Vazyme, Nanjing, China) according the manufacturer’s instruction. Luc-Meis2 ^3’ UTR-^^WT^, Luc-Meis2 ^3’ UTR-^^MUT^, or PMIR ^vector^ were co-transfected into GC cells using Jet transfection reagent. In addition, the promoter sequences of HMGB3 and the truncated sequence of HMGB3 were cloned into the pGL3 vector. Then, mutating the potential Meis2 binding site and insert it into the pGL3 promoter vector. After 48 h, cells were harvested and lysed, following the experimental steps in the Dual Luciferase Reporter Assay Kit (Vazyme, Nanjing, China). The relative luciferase activity is reckoned by measuring the ratio of firefly luciferase activity and Renilla luciferase activity.

### RNA isolation and extraction and real time quantitative PCR(RT-qPCR)

According to standard recommendations, we used the TRIzol Kit (15,596,026, Thermofisher, USA) to isolate and extract total RNA from GC cell lines. Complementary DNA (cDNA) was synthesized according to the instructions of the reverse transcription MMLV kit (Vazyme, Nanjing, China). RT-qPCR result analysis were shown as we mentioned before. The results of RT-qPCR experiments were used to calculate the relative expression levels of different genes using the 2^−ΔΔCt^ method [32].

### Western-blot

The detailed experimental procedure of total protein extraction and detection were performed as referred Shanghai Epizyme Biological Technology Corporation protocol. Antibodies against ALDH1A1 (1:1000, proteintech), OCT3/4 (1:3000, proteintech), SOX2 (1:2000, proteintech), Meis2 (1:1000, CST), HMGB3 (1:1000, CST), GAPDH (1:1000, proteintech) were employed in this research [33].

### Flow cytometry

MKN-45 ^miR−18-^^oe^, MKN-45 ^miR−18-^^kd^, AGS ^miR−18-^^oe^, AGS ^miR−18-^^kd^ and vector cells were harvested and washed three times with PBS and stained with anti-CD44 (750,211, BD Biosciences). The flow cytometer (FACSymphony™ A1, BD Biosciences) was used for determination of CD44^+^ sub-population. The results of the experiment were analyzed through flow cytometry software as previously described [34].

### Spheroid formation assay

The Spheroid formation medium mainly includes 0.48 μg/mL hydrocortisone (ab141250, abcam, USA), 4 μg/mL heparin (ab270804, abcam, USA) and the MammoCult Proliferation Supplements (99–603-CV, corning, USA). The prepared medium was transferred on a 24-wells ultra-low attachment spheroid microplate (Corning costar, Jiangsu, China). Each group had three parallel experiments. Then, 3000–5000 cells/well were added to Spheroid formation medium. A number of spheroids were recorded and taken pictures under a microscope after 10 days [35].

### Chromatin immunoprecipitation assay

Chromatin Immunoprecipitation (ChIP) was performed using Thermo Fisher Chromatin Immunoprecipitation Kit (Cat. No. PA5-82,072, Thermo) follow the manufacturer’s experimental procedures. Primers for Meis2 binding sites on the promoter of HMGB3 (sense: 5’- ggatcttccagagatAGTTTGTAGTTCAGCTTCAGGCCA −3’; antisense: 5’- ctgccgttcgacgatTCTGCTCTCCTGACACACCCTAG −3’) was used for RT-qPCR assay [36]. The detailed information about ChIP-qPCR primers are shown in Table 1.

**Table 1.** Sequences of primers used for ChIP qRT-PCR in this study

Name		Sequences
miR-18	Forward (5’-3’)	CTCCTTTGTGTTTATGAGAGACCTG
	Reverse (5’-3’)	ATGTTTTCCCATTTGTTTGTGTTAT
Meis2	Forward (5’-3’)	ggatcttccagagatACACACACGCCTTTGGCTACA
	Reverse (5’-3’)	ctgccgttcgacgatTTTTGGAGAACATAAGCAATTTTATTC

### RNA-FISH assays

Fluorescence in situ hybridization assay was utilized to analyze the expression of miR-18 in GC cells (AGS, MKN45 and MGC803) and GES-1 (Human gastric mucosal epithelial cells). Three parallel experiments were repeated for each group. Cells were permeabilized with pre-cooling 0.1% Triton X-100, then slides were fixed in 4% paraformaldehyde (Sigma). Then cells were washed three times by prehybridization buffer and then discarded, and the miR-18 probe was hybridized overnight. The next day, after washing with SCC buffer, cells were stained with DAPI and tested for fluorescence with a laser scanning confocal microscope [37].

### In vivo *tumorigenic research*

The mice were operated and housed according to the protocols approved by the Ethics Committee of Xiamen University (2021-SR-322). Before the assays, cyclophosphamide (MedChemExpress USA) was infused from the tail vein of nude mice for 3 consecutive days to suppress the immune system of nude mice. Then, the nude mice were segmented into three groups: NC group, miR-18 overexpression group, and miR-18 knockdown group. Nude mice in each group were injected at a density of 1 × 10^6^ (high concentration group), 1 × 10^5^ (medium concentration group), and 1 × 10^4^ (low concentration group) cells/tumor, respectively. After 8–10 days, the nude mice were anesthetized with isoflurane for euthanasia and tumor separation. The ratio of stem cells was calculated using online website (http://bioinf.wehi.edu.au/software/elda/) ELDA (Extreme Limiting Dilution Analysis) [38].

### Bioinformatics analysis

LinkedOmics was used to identify the correlation between miR-18 and ALDHA1, sox2, and oct4 genes in the TCGA CESC database (http://www.linkedomics.org/login.php). Prediction of miR-18 target genes using online website starbase, DIANA, miRmap, TargetScan, and miRanda.

### Statistical analysis

GraphPad Prism 5.0 software (GraphPad Software, CA, USA) was used to complete statistical analysis. Unpaired Student’s t-test or one-way ANOVA (analysis of variance) was performed to analyze discrepancies between groups. Statistical analyses were carried out by *Student’s t* test. P values <0.05 was considered as statistically significant.

## Result

In this study, we aimed to investigate the effects and underlying mechanisms of miR-18 on gastric cancer stemness. The role of miR-18 as an oncogene was initially evaluated through TCGA database analysis in the development of GC. Subsequently, RNA-FISH, Flow cytometry, Western blotting, and RT-qPCR were applied to analyze the biological function of miR-18 in gastric carcinogenesis. The miRNA database was used to search potential downstream targets of miR-18, and Meis2 was found to have the highest score. Moreover, CHIP experiments found that Meis2 was significantly enriched in HMGB3 promoter. Collectively, our study demonstrated that MiR-18 promoted gastric cancer stemness by targeting Meis2 to suppress HMGB3 expression.

### 3.1 miR-18 is highly expressed and hardly affects cell proliferation ability

**Figure 1.**
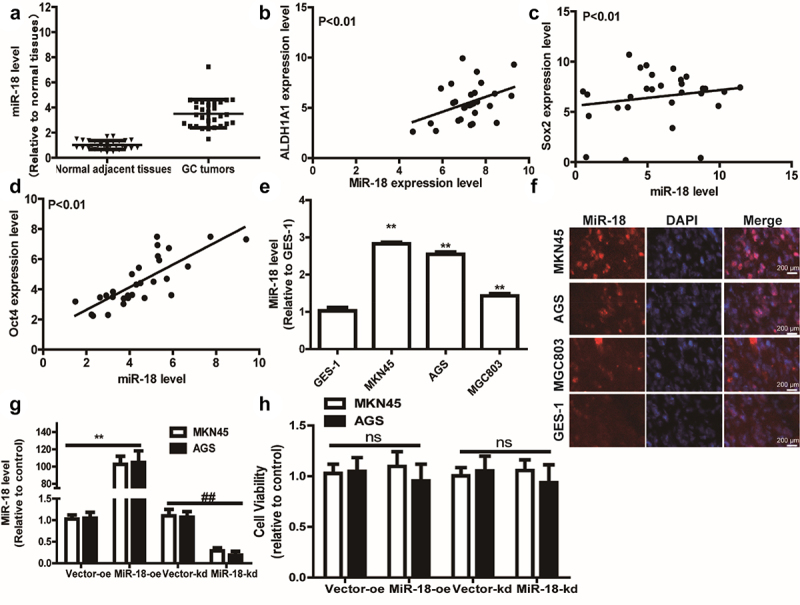
**miR-18 expression is significantly increased in GC tissues and exhibits a positive correlation with the stemness markers**. (a) TCGA database analysis found that miR-18 is highly expressed in gastric cancer tissues compared to that in normal adjacent tissues. **(B, C and D)** Pearson correlation analysis found that the expression of miR-18 was negatively correlated with ALDHA1, sox2, and OCT. (e) RT-qPCR was applied to detect miR-18 level in GES-1, MKN45, AGS, MGC803 gastric cancer cell lines. (f) The expression levels of miR-18 in GES-1, MKN45, AGS, and MGC803 cells were analyzed by RNA-FISH assay. (g) The expression level of overexpression/knockdown miR-18 was detected by RT-qPCR. (h) The influence of overexpression/knockdown of miR-18 was tested on the viability of gastric cancer cells by CCK8. Analyzed using t-test in GraphPad, data were performed as mean ± SD, n ≥ 3, *p < 0.05, **p < 0.01, ***p < 0.001 vs control.

Previous studies had shown that miR-18 was highly expressed in several types of cancers and was negatively correlated with the patient’s prognosis [39]. Firstly, we utilized the bioinformatics analysis of GC data from the Cancer Genome Atlas (TCGA) to examine that compared with adjacent tissues, miR-18 level was upregulated in GC tissues (Figure 1a). Furthermore, we explored the expression correlation between some typical stemness markers (ALDH1A1, SOX2, OCT4) and miR-18 across GC cell line using R2: Genomics Analysis and Visualization Platform. Interestingly, we found that miR-18 level was positively correlated with the expression of ALDH1A1, SOX2, OCT4 (Figure 1b-d). Then, **RT-qPCR** was carried out to measure miR-18 level in GC cells and normal gastric epithelial cells and we found that miR-18 level was remarkably higher in GC cells than that in GES-1 cells (Figure 1e). Moreover, RNA-FISH assay was used to analyze the expression of miR-18 in GC cell lines (AGS, MKN45 and MGC803) and GES-1 (human gastric epithelial cells). The result is shown in figure 1f, miR-18 is highly expressed in GC cell lines and low in human gastric epithelial cells. Then, we constructed GC cells with or without miR-18-stable knockdown or overexpression using lentivirus infection and confirmed the infection efficiency (Figure 1g). Additionally, the effects of miR-18 on the cell proliferation ability were determined and it was found that miR-18 hardly affected the cell proliferation ability of GC cells (Figure 1h). The above results indicate that miR-18 acts as an oncogene in GC cells and is closely related to the stemness of GC cells.

### 3.2 *MiR-18 promotes the stemness of GC cells* in vitro

**Figure 2.**
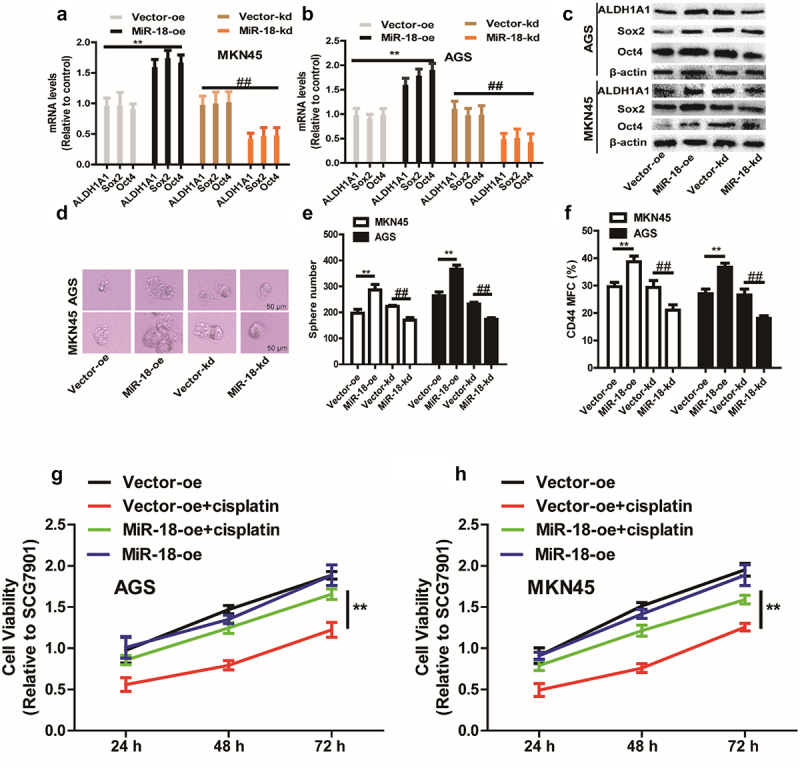
**The effect of miR-18 on the stemness of gastric cancer cells**. (a and b) **RT-qPCR** was used to detect the expression of stemness markers (ALDHA1, SOX2, OCT4) in MKN45 and AGS cells with miR-18 overexpression or knockdown. (c) Western blotting was performed to examine the expression of the stem cell markers ALDHA1, SOX2, OCT/4 in the cells depicted in (a). (d and e) The sphere-formation assays were carried out to determine the size and number of spheres in the cells depicted in **(A)**. (f) Flow cytometry was constructed to evaluate the CD44^+^ subpopulation in the cells depicted in **(A)**. (g and h) CCK8 assay was performed to estimate the cell viability in the cells depicted in **(A)** with or without cisplatin treatment. Analyzed using t-test in GraphPad, data were performed as mean ± SD, n ≥ 3, *p < 0.05, **p < 0.01, ***p < 0.001 vs control.

We first inspected the effects of miR-18 on GC cell stemness, and discovered that the stemness markers (ALDH1A1, SOX2, OCT4) expression was upregulated in miR-18 overexpressed cells and downregulated in miR-18 knockdown GC cells (Figure 2a **–** c). To further examine the effect of overexpression of miR-18 on GC cells with stemness. We also explored whether miR-18 has impact on sphere-formation ability and outcropped that both the dimension and quantity of spheres were increased in miR-18-overexpressed cells and decreased in miR-18-knockdown cells (Figure 2d and 2e). Furthermore, the CD44^+^ subpopulation with the stemness characteristics was increased by miR-18 overexpression (figure 2f). Since cancer stemness is related to chemoresistance, we performed a cell viability assay to detect the effects of miR-18 on the chemotherapeutic sensitivity of GC cells. As shown in Figure 2g and 2h, overexpression of miR-18 resulted in some extent of resistance to cisplatin.

### 3.3 *miR-18 promotes tumor initiation of gastric cancer cells* in vivo

**Figure 3.**
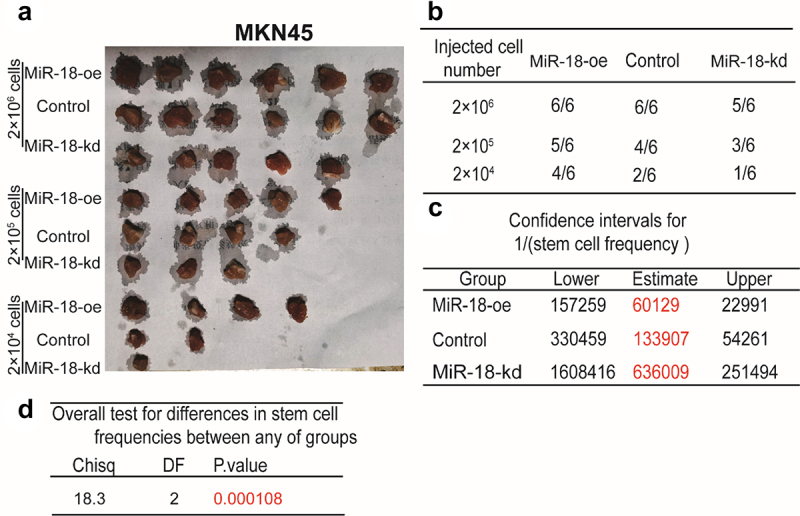
MiR-18 promotes the tumor-initiating ability of gastric cancer cells *in vivo*. **(A and B)** Tumor images of GC cells with miR-18 overexpression or knockdown. (B) The injected cell number of gastric cancer were selected and formation ratio was calculated. **(C and D)** The confidence intervals for 1/(stem cell frequency) was evaluated.

To investigate the role of miR-18 in regulating the stemness of GC cells, the tumor-initiating ability was assessed in MKN45 cells with miR-18 overexpression or knockdown. *In vivo* experiments combined limiting dilution analysis were performed in GC cells with miR-18 ectopic expression. As shown in Figure 3a **and** 3b, tumors sprung from miR-18-overexpressed cells performed a significant increase of formation rate, while miR-18 knockdown cells exhibited an opposite effect. Furthermore, the confidence intervals for 1/(stem cell frequency) was increased or decreased, respectively, in cells with miR-18 overexpression or knockdown through limiting dilution analysis (http://bioinf.wehi.edu.au/software/elda/) (Figure 3c). Moreover, a significant difference of colligation test for differences in stem cell frequencies was revealed between any of groups (Figure 3d). The above results indicate that miR-18 can promote the stemness of GC cells.

### 3.4 Meis2 is identified as the direct target of miR-18 target

In this section, we investigated the mechanism by which miR-18 facilitates GC cells stemness. Through three online database predictions (Targetscan, miRDB, miRwalk), we found that Meis2, with the highest score, was predicted as a potential target of miR-18. Therefore, Meis2 was chosen for subsequent mechanism research.

**Figure 4.**
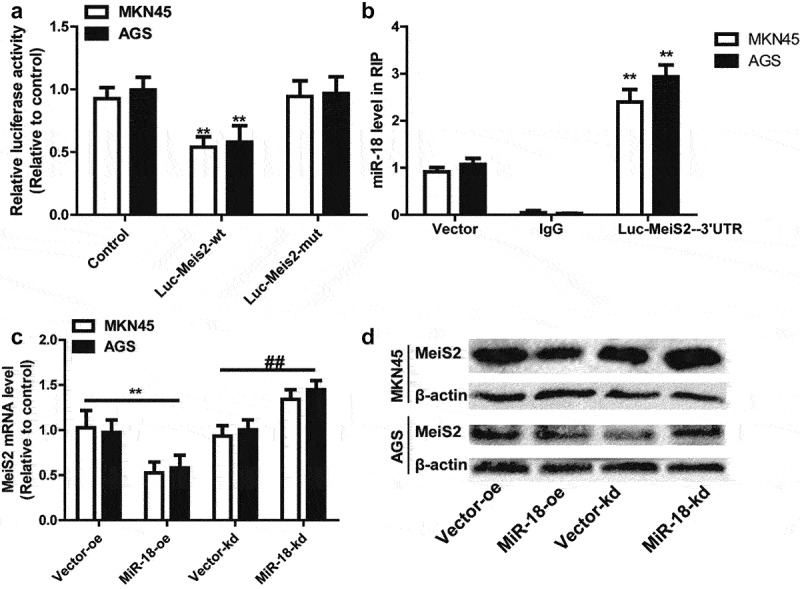
**MiR-18 directly binds to Meis2^3^^’^^UTR^**. (a) Schematic diagram showing the binding of miR-18 and meis2 by luciferase reporter method. (b) RNA immunoprecipitation assay was performed to confirm the binding of miR-18 on Meis2. (c and d) RT-qPCR and WB were performed to detect the mRNA and protein levels of Meis2 in GC cells with miR-18 overexpression or knockdown. Analyzed using t-test in GraphPad, data were performed as mean ± SD, n ≥ 3, *p < 0.05, **p < 0.01, ***p < 0.001 vs control.

To further verify that miR-18 targets Meis2 to promote the stemness of gastric cancer cells, we chose the luciferase reporter assay for confirmation. Luc-Meis2^3’ UTR-^^WT^ or Luc-Meis2^3’ UTR-^^MUT^ sequences and miR-18 binding site mutant or not were subsequently amplified and sub-cloned into the luciferase reporter vector. Luciferase activity of Luc-Meis2^3’ UTR-^^WT^ was remarkably suppressed by miR-18 overexpression but the Luc-Meis2^3’ UTR-^^MUT^ luciferase activity had little change significantly (Figure 4a). In addition, in PMIR-Meis2^3^^’^^UTR-^^WT^ or PMIR- Meis2^3^^’^^UTR-^^MUT^ overexpressing cells, the miR-18 complex was pulled down by Ago2 in Meis2^3^^’^^UTR-^^WT^ and Meis2^3^^’^^UTR-^^MUT^ overexpressing cells, and RT-qPCR detection was found that the expression of miR-18 was enriched in Meis2-3’ UTR-WT cells, but decreased in Meis2-3’ UTR-mut overexpressing cells (Figure 4b). Moreover, Western blot and RT-qPCR were detected the effect of overexpression or knockdown of miR-18 on Meis2 in MKN45 and AGS cells. As shown in Figure 4c **and** 4d, overexpression of miR-18 can reduce the expression of Meis2 at the mRNA and protein levels. Knockdown of miR-18 exerted opposite effects. Overall, these results prove that miR-18 can directly bind to Meis2 ^3’UTR^ in GC.

### 3.5 MiR-18 facilitates the stemness of GC cells though targeting Meis2

**Figure 5.**
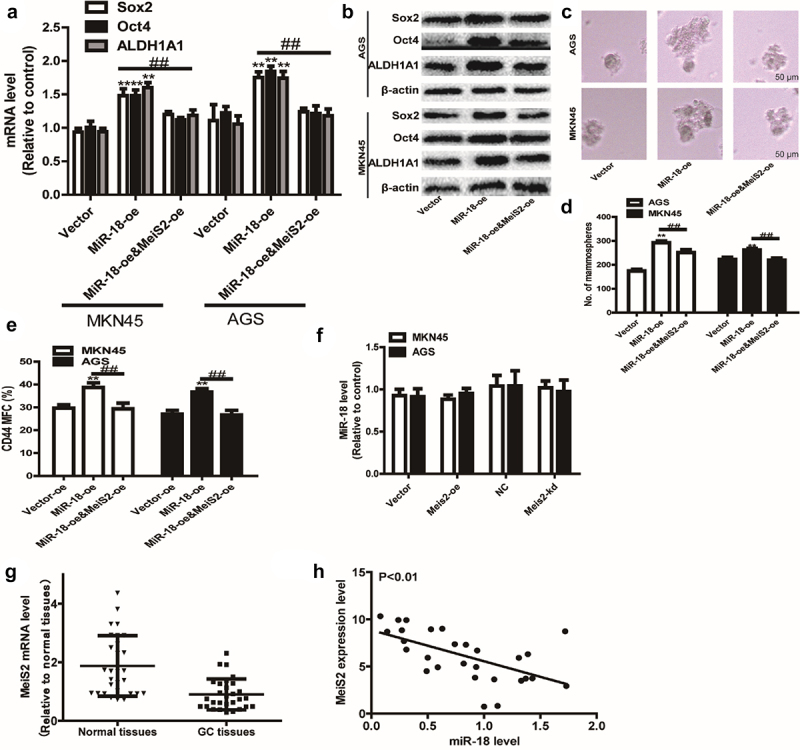
(a and b) RT-qPCR and WB were performed to the expression of stemness marker in GC cells with miR-18 overexpression plus Meis2 overexpression or not. (c and d) Sphere-forming assays were constructed to examine the sphere size and number of GC cells with miR-18 overexpression plus Meis2 overexpression or not. (e) Flow cytometry was performed to detect CD44+ subpopulation in the cells described in (c). (f and g) The online database were used to analyze the expression level of Meis2 in normal tissues and GC tissues, as well as the correlation between miR-18 and Meis2, and the results were shown. Analyzed using t-test in GraphPad, data are performed as mean ± SD, n ≥ 3, *p < 0.05, **p < 0.01, ***p < 0.001 vs control.

Then, we speculated whether miR-18 can promote the stemness of GC cells by targeting Meis2. Western blot and RT-qPCR were used to detect the effect of miR-18 overexpression, overexpression of miR-18 and overexpression of Meis2 on the stemness of GC cells. The results of RT-qPCR and western blotting indicated that overexpression of miR-18 could promote the expression of gastric cancer cell stemness markers at the mRNA and protein levels. However, overexpression of meis2 attenuated the promoting effect of miR-18 on the expression of stemness markers in GC cells (Figure 5a **and** 5b). Moreover, Meis2 overexpression attenuated miR-18-induced reduction of mammary spheroid formation ability (Figure 5c **and** 5d). Furthermore, flow cytometry was performed to detect the ratio of CD44^+^ subpopulation. As shown in Figure 5e, overexpression of Meis2 can attenuate the effect of miR-18 on the ratio of CD44^+^ subpopulation. We then explored the effect of overexpression or knockdown of meis2 on miR-18 expression levels. The results are shown in figure 5f, overexpression or knockdown of Meis2 had no effect on miR-18 levels. In addition, the expression of Meis2 was analyzed in GC tissues and adjacent tissues from the TCGA database. The results showed that Meis2 was lowly expressed in GC tissues and highly expressed in normal tissues (P < 0.001) (Figure 5g). Finally, we analyzed the correlation between miR-18 and Meis2 through an online database and found that they were negatively correlated (Figure 5h). Overall, these results indicate that miR-18 promotes the stemness of GC cells by targeting Meis2, thereby inhibiting the occurrence and development of GC cells.

## 3.6 miR-18 modulates HMGB3 expression by directly targeting Mesi2

A recent study reported that the miR-181 cd/Meis2/Hmgb3 is involved in the conversion of IT to MLP phenotype [26]. HMGB3 has been shown to be involved in regulating gene transcription, replication, recombination, DNA repair, and genome stability, and as an oncogene, high expression of HMGB3 is related to the invasion, metastasis and poor prognosis of various tumors, but the mechanism in GC is still confusing [40,41]. Therefore, we speculate whether miR-18 directly targets Mesi2 to regulate the expression of HMGB3 to promote the stemness of GC cells.

**Figure 6.**
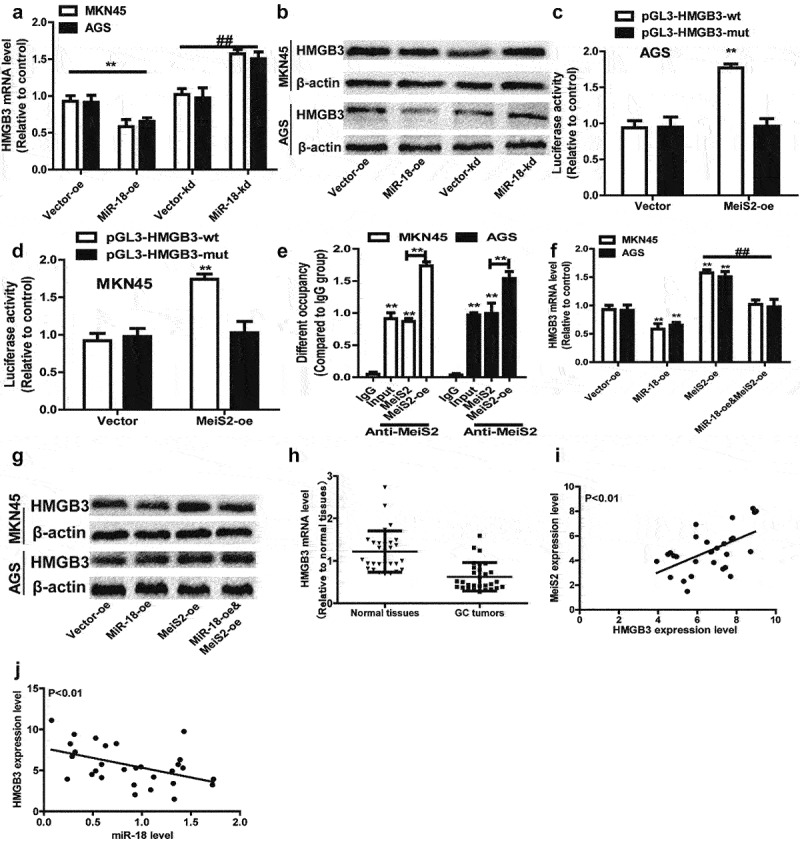
(a and b) RT-qPCR and WB were performed to detect the expression level of HMGB3 in AGS and MKN45 cells with miR-18 overexpression or knockdown. (c and d) The luciferase reporter method was used to detect the impact of meis2 overexpression on the fluorescence intensity of HMGB3-wt and HMGB3-mut. (e) CHIP-qPCR was verified the binding between the transcription factor Meis2 and the promoter HMGB3. (f and g) RT-qPCR was used to examine HMGB3 expression in GC cells with miR-18 overexpression plus Meis2 overexpression or not. **(H, I and J)** The online database were used to analyze the expression level of HMGB3 in normal tissues and GC tissues, as well as the correlation between HMGB3 and Meis2/miR-18, and the results are shown in the figure. Analyzed using t-test in GraphPad, data were performed as mean ± SD, n ≥ 3, *p < 0.05, **p < 0.01, ***p < 0.001 vs control.

We measured the effect of miR-18 on the expression of HMGB3 in MKN45 and AGS cells by Western blot and RT-qPCR to verify the hypothesis that miR-18 promotes GC cells by regulating HMGB3. The results are shown in Figure 6a and 6b, overexpression of miR-18 down-regulated the expression of HMGB3 at mRNA and protein levels. When miR-18 was knocked down, the result was opposite. In order to confirm the interaction between Meis2 and HMGB3, the luciferase reporter assay was used to verify that overexpressed Meis2 can bind to the promoter of HMGB3 to regulate the stemness of GC cells. The result is shown in Figure 6c **and** 6d, which revealed the luciferase activity of HMGB3 ^PGL3-^^WT^ was remarkably activated by Meis2 overexpression, while no significant changes in the activity of HMGB3 ^PGL3-^^MUT^ was perceived. Then we explored the mechanism by which Meis2 regulates HMGB3 to inhibit the stemness of GC cells. We performed ChIP-qPCR and found that HMGB3 was enriched in DNA pulled down by anti-Meis2, and Meis2 overexpression increased the enrichment of HMGB3 (Figure 6e). Moreover, we found that overexpression of miR-18 can inhibit the expression of HMGB3, but overexpression of Meis2 can reverse the inhibitory effect of miR-18 on HMGB3 in mRNA and protein level (figure 6f **and** 6g). Finally, we analyzed the expression of HMGB3 in tumor tissues and adjacent tissues from the TCGA database. The result is shown in Figure 6h, HMGB3 is lowly expressed in tumor tissues, while highly expressed in normal tissues. Notably, we found that, in the TCGA database, the expression of Meis2 and HMGB3 was positively correlated, and the expression of miR-18 and HMGB3 was negatively correlated (Figure 6i **and** 6j). Collectively, these results demonstrate miR-18a directly targets Mesi2 to regulate the expression of HMGB3 to promote the stemness of GC cells.

## 4. Discussion

GC is a common malignant tumor, the global incidence rate varies greatly, and it seriously threatens human life and health [42]. Therefore, the identification of reliable GC diagnostic markers is still an important research focus. Previous studies have shown that miR-18, a member of the miR-17–92 cluster, as a potential oncogene, is highly expressed in various cancer tissues including pancreatic cancers [43], hepatocellular carcinoma [44], colorectal carcinomas and primary squamous cell lung carcinoma [45]. As a key regulator of gastric cancer, miR-18 may promote the progression of GC. Therefore, early detection of the expression of miR-18 plays a key role in improving the prognosis of GC. In current studies, we keep a watchful eye on to miR-18 and explored its role and mechanism in GC.

CSCs are a small group of heterogeneous cells that exist interior tumor cells and have tumor-initiating capacity. Under the influence of certain environments, CSCs can drive the metastasis and recurrence of tumor populations. Moreover, Yan Chen et al. demonstrated miR-18a regulates the expression of P53 by targeting IRF2 in patients with GC, thereby promoting the migration, invasion and metastasis of GC cells [17]. Since CSCs contribute to metastasis and recurrence, we assume that miR-18 can positively regulate the stemness of GC cells. Indeed, RNA sequencing results found that miR-18 has a significant positive correlation with the stemness of GC cells which is confirmed by the enhancement of the sphere-forming ability, the up-regulation of the ratio of CD44^+^subpopulations with stemness, increased expression of stemness markers, and the tumor-initiating capability. Furthermore, many researches have manifested that up-regulation of miR-18 can promote proliferation in HCC both *in vitro* and *in vivo*; Conversely, inhibition of miR-18 significantly decreased the xenograft tumor volume and weight for both HCC cell lines [46]. Notably, JingZhang et al. have been reported that lncRNA SNHG15/miR-18a-5p axis promotes cell proliferation in OC through active Akt/mTOR signaling pathway [47]. These phenomena are in keeping with our results that miR-18 enhances the stemness and chemotherapy resistance in GC cells.

To investigate the detailed mechanism of miR-18 in the progression of GC, bioinformatics and luciferase reporter gene detection indicate that miR-18 directly targets Meis2, thereby regulating the expression of downstream targets. Meis2, a transcription factor, is a member of the Meis protein family that plays a vital role in regulating cell fate during cell proliferation and is related to the pathogenesis of human cancer [48,49]. For example, Meis2 has been revealed to be overexpressed in ovarian cancer and inhibit the proliferation and tumorigenicity of ovarian cancer cells, which was connected to improving the prognosis for patient with ovarian cancer [50]. Depletion of Meis2 resulted in increased tumor growth over time *in vivo*, and was associated with increased expression of the tumorigenic genes cMYC and CD142 [48]. Therefore, we speculate that this miR-18/Meis2 axis is a common phenomenon in GC. In the present study, the overexpression of Meis2 can attenuate the stemness of GC cells, which was characterized as the alteration of stem cell markers and the ratio of CD44^+^ subpopulation with stemness, as well as the size and number of GC cells. Finally, we found that HMGB3 is involved in stemness signal pathway regulation through GO enrichment and GSEA analysis of TCGA database. Moreover, Jun Gu et al. study found HMGB3 is highly expressed in tumors as an oncogene. Overexpression of HMGB3 enhanced spheroid formation as well as upregulated the expression of Nanog, SOX2 and OCT-4 genes/proteins. Inversely, HMGB3 silence decreased CD44^+^/CD24^−^ subpopulation with stemness in breast cancer cells [51]. Therefore, we speculate whether miR-18 promotes the stemness of GC cells by targeting Meis2 to up-regulate the expression of HMGB3 in gastric cancer. We found that miR-18 and HMGB3 are negatively correlated. Overexpression of miR-18 or knockdown HMGB3 can promote the stemness of GC cells.

There was some inadequacy in this study. The functions of Meis2 and HMGB3 are carried out through multi-step bioinformatics methods, and needed to be further confirmed in our future research through in *vitro* and *in vivo* experiments; however, these data provide clues for studying the mechanism of the miR18/Meis2 axis regulating HMGB3 to promote the stemness of GC cells.

## Conclusion

As far as we know, this is the first time revealing that miR-18 facilitates the stemness of GC by downregulating HMGB3 though targeting Meis2. This research may provide a strategy for the clinical treatment of GC, especially by regulating the stemness closely related to tumors.
